# Perinatal Health Indicators (PHI) Data Tool

**DOI:** 10.24095/hpcdp.44.7/8.07

**Published:** 2024-07

**Authors:** Stephanie Metcalfe, Jennifer Lye, Hongbo Liang, Holly Arscott, Chantal Nelson, Wei Luo

**Affiliations:** 1 Center for Surveillance and Applied Research, Health Promotion and Chronic Disease Prevention Branch, Public Health Agency of Canada, Ottawa, Ontario, Canada

The Maternal and Infant Health Section of the Public Health Agency of Canada (PHAC) is pleased to announce an update to the Perinatal Health Indicators (PHI) Data Tool. 

The interactive Data Tool on the PHAC Infobase website presents statistics on maternal, fetal and infant health in Canada based on data from the Canadian Institute for Health Information’s (CIHI) Discharge Abstract Database (DAD), the Canadian Community Health Survey (CCHS), and the Canadian Vital Statistics (birth, stillbirth and death databases). 

The data include 20 indicators grouped into four key health domains: health behaviours and practices, health services, maternal outcomes, and infant outcomes. For this update, five new indicators were added and three existing ones were modified.

To access the latest Perinatal Health Indicators Data Tool, visit https://health-infobase.canada.ca/phi/ .

